# Expanding the Phenotypic Spectrum of FOXC1-Related Axenfeld-Rieger Syndrome Type 3: A Case Report

**DOI:** 10.7759/cureus.98380

**Published:** 2025-12-03

**Authors:** Amia Mourad, Harry Ward, Nicholas Lorenz, Krishna Patel, Madhura Butala

**Affiliations:** 1 Medicine, Lake Erie College of Osteopathic Medicine, Jacksonville, USA; 2 Orthopaedic Surgery, Lake Erie College of Osteopathic Medicine, Bradenton, USA; 3 Pediatrics, Ascension St. Vincent's, Jacksonville, USA

**Keywords:** anterior segment dysgenesis, axenfeld–rieger syndrome, coloboma, congenital heart disease (chd), corectopia, craniofacial dysmorphism, foxc1 deletion, posterior embryotoxon, posterior fossa malformation, sensorineural (sn) hearing loss

## Abstract

Axenfeld-Rieger syndrome (ARS) is an inherited disorder that commonly affects ocular and other systemic structures. We introduce a case of a male infant who presented to the clinic after genomic microarray testing discovered a chromosomal deletion involving the FOXC1 gene. The patient was found to have anterior segment anomalies, craniofacial differences, auditory insufficiency, and other systemic findings such as an atrial communication defect throughout the first two years of life. Genetic testing performed shortly after birth supported the diagnosis of ARS as the cause of these various findings. This early recognition of ARS permitted prompt initiation of multidisciplinary care and allowed the patient to receive the appropriate ophthalmologic, cranial, renal, cardiac, and developmental testing to provide sufficient care and future monitoring. This case highlights the importance of genetic evaluation and ongoing coordinated care to anticipate and manage the diverse features of patients with ARS.

## Introduction

Axenfeld-Rieger syndrome (ARS) is a rare autosomal dominant disorder with a prevalence of approximately 1:200,000 and is categorized as an anterior segment syndrome [1,2]. The Axenfeld anomaly was originally described by Theodor Axenfeld after he observed a posterior embryotoxon with adhesion of peripheral iridal strands. The Rieger anomaly was later described by Herwig Rieger as a combination of a posterior embryotoxon, iris hypoplasia, polycoria, and corectopia [3]. In contemporary usage, these two anomalies are combined to describe ARS, which is now understood to include systemic findings as well as the historical ocular findings [1]. Anterior segment syndromes are part of a spectrum of disorders that include the iris, cornea, and lens, and are the leading cause of ocular morbidity [4]. Specifically, Axenfeld-Rieger anomaly (ARA) defines a combination of ocular abnormalities including iris hypoplasia, posterior embryotoxon, and either or both of corectopia and iridocorneal adhesions [5,6].

ARS is characterized by the combination of ARA with systemic irregularities involving cardiac, skeletal, and auditory tissues, as well as other physiologic and neurodevelopmental abnormalities. Patients with ARS are also at risk of developing glaucoma due to abnormal formation of the anterior segment, requiring lifelong ophthalmologic surveillance [1]. It can be further broken down into three groups depending upon the genes involved, with PITX2 and FOXC1 being widely recognized as the two most commonly involved genes resulting in ARS type 1 and 3, respectively. PITX2 and FOXC1 are involved in approximately 40% of all cases of ARS, and may result in ARA with or without systemic findings. In cases of ARS, the systemic findings seen in mutations involving PITX2 tend to be associated with dental and umbilical anomalies, while those involving FOXC1 are more likely to involve cardiac or auditory systems [2,5].

Both ARS type 1 and ARS type 3 are autosomal dominant; ARS type 1 is associated with heterozygous mutations of PITX2 on chromosome 4q25, while ARS type 3 is associated with heterozygous mutations of FOXC1 on chromosome 6p25 [1]. Our case presents a male infant who presented with potential prenatal signs of ARS, which were confirmed with immediate postnatal imaging and genetic testing that showed an interstitial deletion on chromosome 6 within band 6p25.3, including the genes FOXC1 and GMDS.

## Case presentation

A male infant was born at term following a pregnancy notable for several prenatal imaging abnormalities. Prenatal fetal magnetic resonance imaging (MRI) revealed prominent posterior fossa fluid communicating with the fourth ventricle and mild cerebellar vermis hypoplasia, suggestive of a Blake’s pouch cyst. Prenatal ultrasound revealed echogenic bowel and bilateral pyelectasis with mild ureteral dilation.

**Figure 1:**
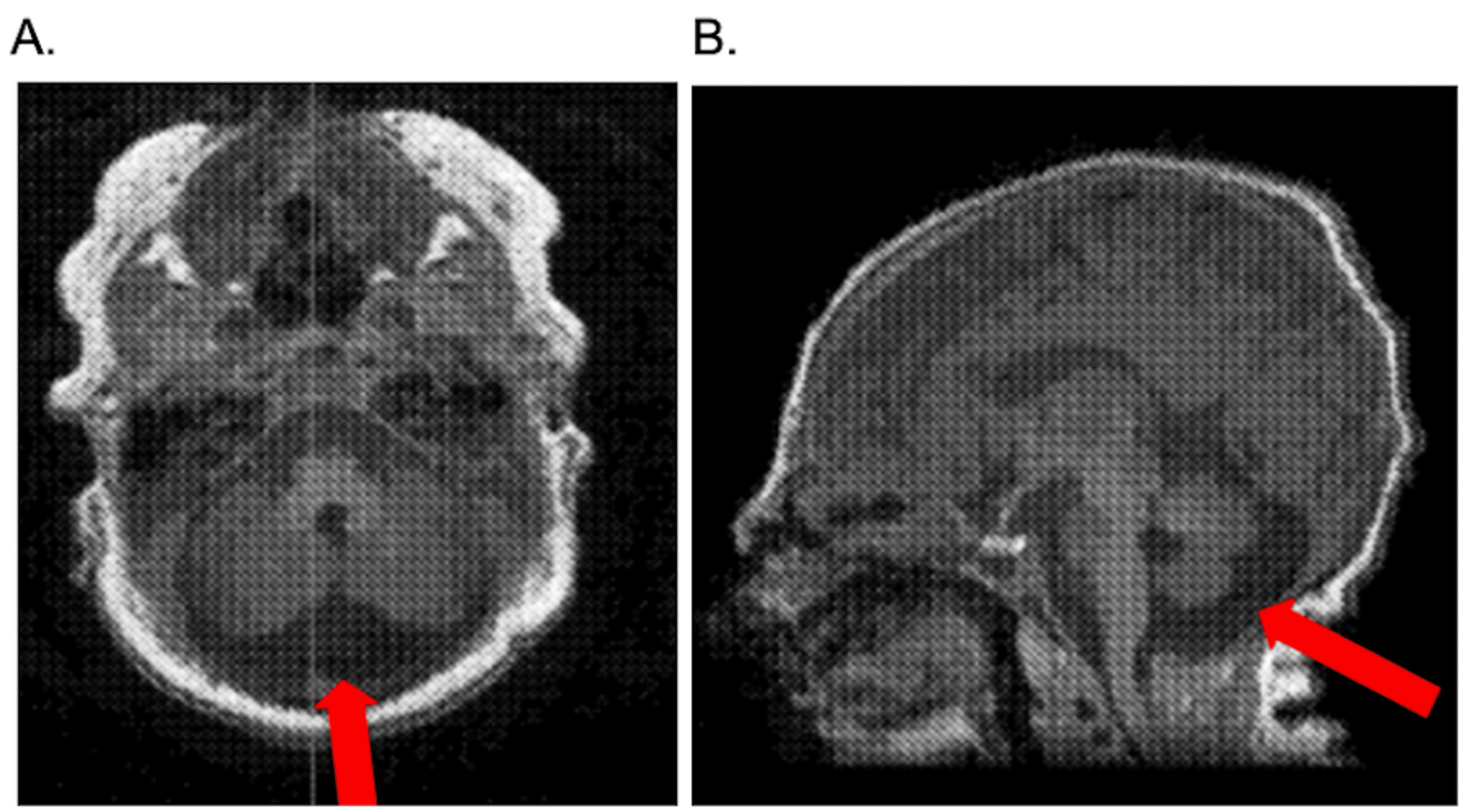
Brain MRI without contrast demonstrating mild prominence of the posterior fossa cerebrospinal fluid. (A) Axial view. (B) Sagittal view.

After birth, the infant exhibited dysmorphic features, including a wide glabella, hypertelorism, bitemporal narrowing, and a flat nasal bridge. Oral anomalies, including a lip tie and a mild tongue tie, were noted, contributing to early feeding difficulties. Postnatal cranial MRI confirmed mild prominence of posterior fossa cerebrospinal fluid without evidence of Dandy-Walker malformation (Figure 1). Additionally, a cranial ultrasound identified a small choroid plexus cyst and a posterior fossa keyhole sign. At hospital discharge, the newborn hearing screen was passed, establishing normal auditory function at that time.

**Figure 2:**
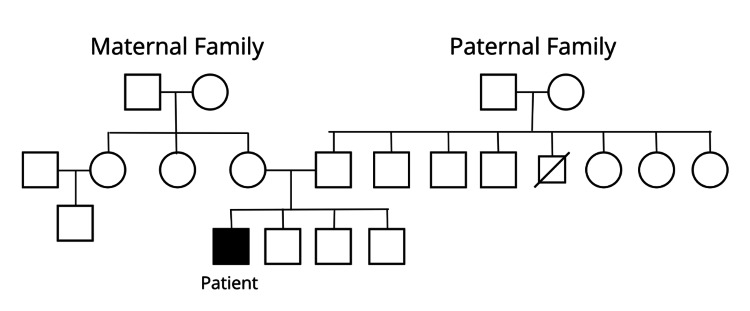
Pedigree showing the proband with Axenfeld-Rieger syndrome. No other family members across three generations are affected, suggesting a de novo pathogenic variant.

Genetic evaluation was performed shortly after birth. Chromosomal microarray analysis identified a pathogenic 1.4 Mb interstitial deletion on chromosome 6p25.3 encompassing FOXC1, GMDS, and at least six other protein-coding genes. FOXC1 is primarily responsible for the patient’s ocular anomalies and posterior fossa findings, consistent with ARS type 3. The loss of the other genes within the deleted region may contribute to additional systemic or developmental features, accounting for variability in the patient’s phenotype beyond the classic manifestations of ARS. Family pedigree analysis (Figure 2) suggests that this deletion is likely de novo, as no other family members are affected. This deletion provides a unifying explanation for the constellation of ocular, craniofacial, neuroimaging, and systemic findings observed in the infant. Genetic counseling was provided to guide family education and long-term care planning.

**Figure 3:**
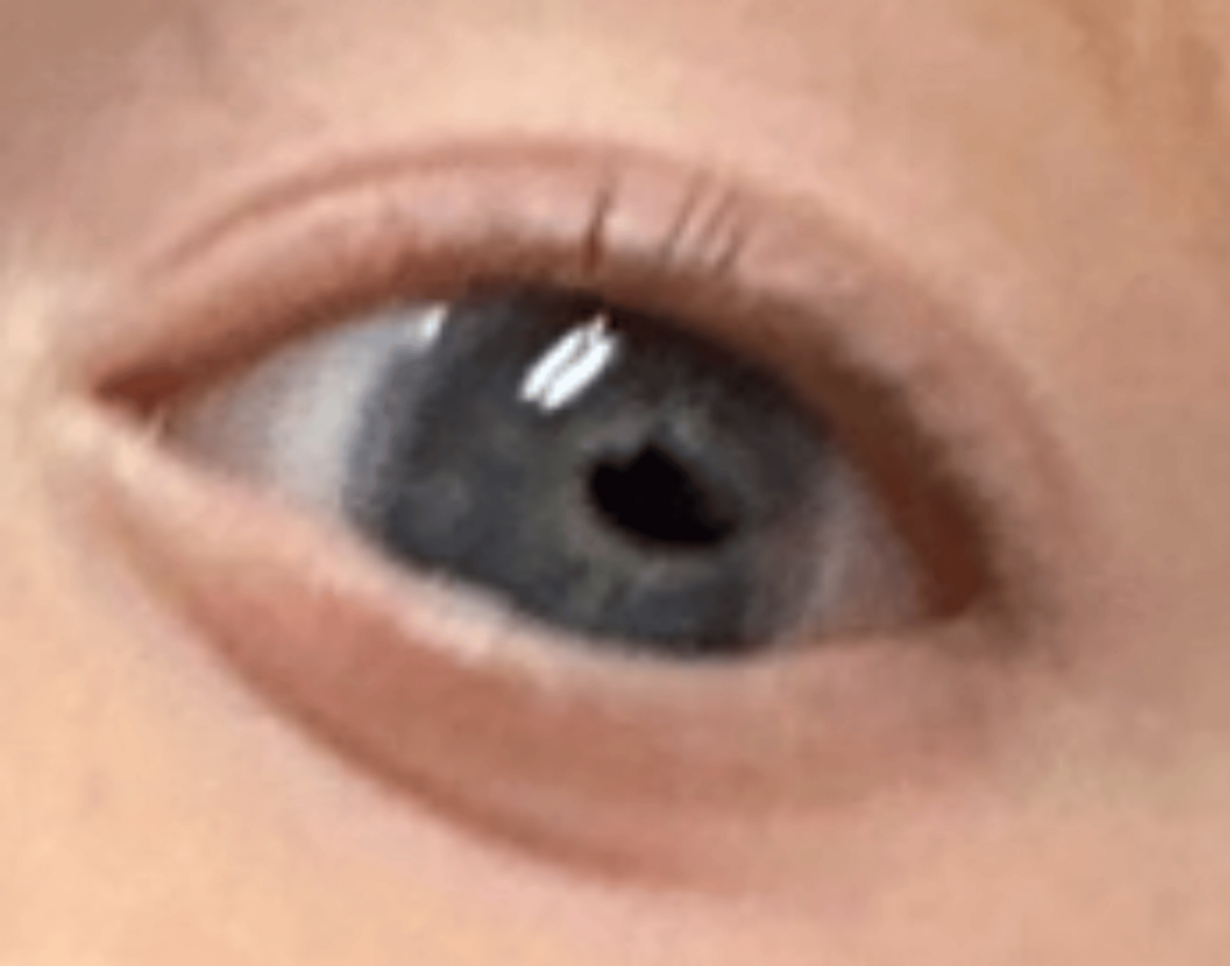
Left eye demonstrating coloboma, cloudy iris, and corectopia, consistent with Axenfeld-Rieger syndrome type 3.

Ophthalmologic evaluation, performed in the first few months of life, revealed anterior segment abnormalities consistent with ARS. Findings included a left-sided coloboma, cloudy irides, posterior embryotoxon, corectopia, and intermittent exotropia (Figure 3). Intraocular pressures were within normal limits. Follow-up ophthalmology visits confirmed continued ocular anomalies with improvement in exotropia control over time, while maintaining a healthy lens and optic nerves.

Systemic evaluations, including renal, cardiac, and growth assessments, were performed in the early months of life. Renal ultrasound demonstrated mild bilateral pelvic fullness and nonspecific bladder debris, which did not require intervention. Cardiac echocardiography revealed a small atrial communication, likely representing either a patent foramen ovale or a small secundum atrial septal defect (ASD), which was hemodynamically insignificant. Growth parameters were below average for his age, raising consideration for potential growth hormone deficiency. The patient was referred to pediatric endocrinology for evaluation, where IGF-1 levels were within the normal range. Developmental milestones were largely appropriate for age, including social smiling, cooing, rolling, and hand-to-midline coordination. The patient did not require treatment at that time and continues to be monitored by endocrinology.

Audiologic assessment was conducted in the first few months of life and initially suggested absent peripheral auditory function in both ears. This finding prompted referral for sedated auditory brainstem response testing, which later demonstrated mild high-frequency (4000 Hz) sensorineural hearing loss. Despite this, responses to speech stimuli in the sound field were within normal limits, and the patient continued to demonstrate age-appropriate auditory responses.

## Discussion

This case highlights the multisystem involvement of ARS associated with a 6p25.3 deletion encompassing FOXC1 and six additional protein-coding genes. Ocular anomalies such as corectopia, posterior embryotoxon, colobomas, and iris clouding are well-established features of FOXC1-related ARS [1,7]. Despite these defects, the patient was visually attentive for his age. However, lifelong follow-up with ophthalmology is crucial to identify any visual deterioration or signs of early-onset glaucoma. This patient also demonstrated subtle cerebellar changes and mild high-frequency sensorineural hearing loss, findings that are less commonly reported [8]. These observations expand the known phenotypic spectrum of FOXC1 haploinsufficiency and suggest a broader developmental role for FOXC1 in hindbrain and auditory system formation.

Systemic manifestations in this patient included craniofacial dysmorphism, mild congenital heart disease, renal anomalies, and growth parameters that were initially concerning but ultimately within normal limits. The deletion of additional genes within 6p25.3 likely contributes to phenotypic variability, reinforcing that contiguous gene deletions can produce a broader array of systemic and developmental features than FOXC1 haploinsufficiency alone [9]. Recognizing this spectrum is clinically important for anticipating potential complications and guiding focused follow-up.

Cardiac anomalies are a recognized feature of FOXC1-related ARS. They range from small, hemodynamically insignificant atrial communications to larger ASDs that may require intervention. In a previously reported patient with a FOXC1 mutation, a congenital ASD was present along with additional systemic anomalies, highlighting the potential severity and variability of cardiac involvement in ARS [10]. In this case, the patient exhibited a small atrial communication that is currently hemodynamically insignificant. Nevertheless, these findings emphasize the importance of ongoing cardiology follow-up to monitor for potential changes and to provide anticipatory guidance.

Neuroimaging initially raised concern for Dandy-Walker malformation due to posterior fossa fluid accumulation and mild cerebellar vermis hypoplasia. Careful review clarified that these changes were not consistent with Dandy-Walker malformation, but they highlight subtle hindbrain anomalies associated with FOXC1 deletions. Previous studies of 6p25.3 deletions have demonstrated that FOXC1 is essential for normal cerebellar development, with affected patients exhibiting a spectrum of posterior fossa anomalies ranging from vermian hypoplasia to cystic changes resembling Dandy-Walker malformation [11]. These findings support FOXC1’s role in hindbrain morphogenesis and emphasize the importance of early and careful neuroimaging evaluation in patients with 6p25 deletions.

Although the patient’s hearing loss was limited to mild high-frequency deficits and auditory responses to speech were within normal limits, this case illustrates the need for ongoing audiologic monitoring in ARS, as FOXC1-related phenotypes can extend beyond the eye [1,2,12,13]. Early genetic diagnosis enables clinicians to anticipate and monitor these variable features, provide family counseling, and coordinate care across multiple specialties. However, this report describes a single patient, which limits generalizability. Because the genetic finding involves a multigene deletion, we cannot definitively attribute specific phenotypic features to the loss of FOXC1 alone versus neighboring genes. Additionally, long-term ophthalmologic and systemic outcomes are not yet available, which restricts our ability to comment on disease progression.

## Conclusions

ARS type 3 due to FOXC1 deletion is a rare condition with significant ocular and systemic manifestations. This case highlights the importance of comprehensive evaluation, as the phenotype may extend beyond the classic ocular findings to include posterior fossa malformations, congenital heart disease, renal anomalies, hearing impairment, and growth disturbances. Recognition of these features facilitates early intervention and coordinated care across multiple specialties, ultimately improving long-term outcomes.
